# Effect of Varying Levels of Dietary Tryptophan on Aggression and Abnormal Behavior in Growing Pigs

**DOI:** 10.3389/fvets.2022.849970

**Published:** 2022-06-01

**Authors:** Maggie Henry, Anna Kate Shoveller, Terri L. O'Sullivan, Lee Niel, Robert Friendship

**Affiliations:** ^1^Department of Population Medicine, University of Guelph, Guelph, ON, Canada; ^2^Department of Animal Biosciences, University of Guelph, Guelph, ON, Canada

**Keywords:** behavior, pig, serotonin, tryptophan, aggression, tail-biting

## Abstract

Body lesions, resulting from tail-biting and ear-biting, can result in decreased health and welfare in pigs. Tryptophan, an indispensable amino acid, is needed to support protein deposition, and the synthesis of the neurotransmitter serotonin, which is important to mood, sleep-wake and eating patterns and might play a role in aggression and abnormal behavior. Two randomized block design studies were conducted to assess the influence of varying dietary tryptophan levels on aggression and abnormal behavior in 8-week-old pigs. Six diets were formulated which met or exceeded all nutrient requirements yet differed according to the dietary tryptophan content. The first study included control (100% standardized ileal digestible tryptophan), supplemented (175% standardized ileal digestible tryptophan), and supplement-plus (250% standardized ileal digestible tryptophan) experimental diets, while the second study included deficient (80% standardized ileal digestible tryptophan), adequate control (105% standardized ileal digestible tryptophan), and extra-tryptophan (130% standardized ileal digestible tryptophan) experimental diets. Concentrations of plasma tryptophan and large neutral amino acids (tyrosine, isoleucine, leucine, valine, and phenylalanine) were analyzed using ultra-performance liquid chromatography and the tryptophan to large neutral amino acid ratio was calculated. Analysis for time active, lying, and engaging in aggressive interactions was carried out using 10-min scan samples to determine behavioral time budgets of the pigs on different experimental diets. Pigs fed diets with supplemented tryptophan had higher concentrations of both plasma tryptophan and tryptophan to large neutral amino acid ratio compared to the pigs fed the control diet (*P* < 0.05) in the first study, while no significant differences were detected for plasma tryptophan or the tryptophan to large neutral amino acid ratio in the second study. Diet did not have an effect (*P* > 0.05) on weight, feed intake or behavior throughout the studies. The results suggest that an increase in dietary tryptophan relative to large neutral amino acids, fed for 29 days, impacts circulating plasma tryptophan and therefore, serotonin concentrations in the pig. Despite an increase in circulating plasma tryptophan in response to an increase in dietary tryptophan in the first study, we failed to see an impact of the dietary treatment on body, tail and ear-biting behavior under the conditions studied.

## Introduction

In modern pig production abnormal and agonistic behavior, including fighting, combative postures, and biting, are typically utilized to gain access to resources, such as food, or to establish a social hierarchy (1); these behaviors can result in injuries, reduced animal welfare and economic loss (2, 3). Abnormal behavior, such as tail-biting (TB), ear-biting (EB), and flank-biting (FB), is detrimental to swine production efficiency and swine welfare and has been hypothesized as a coping mechanism for frustration or ill-health (4–6).

Throughout production, the occurrence and duration of aggression and abnormal behavior may differ based on available resources, such as food, or the phenotype of the pig. Intensively raised pigs are exposed to several stressful stimuli from birth until market due to normal management procedures (7), such as weaning, mixing and transport. Both the biter pig(s) and victim pig(s) experience increased levels of stress and reduced welfare (8, 9), which may increase the monetary costs associated with abnormal behavior. Globally, abnormal behavior leads to millions of dollars in extra costs and lost revenue annually (3). The initiation of abnormal behavior is largely accepted to be multifactorial (10). This multifactorial nature creates difficulties when attempting to accurately predict TB and record TB prevalence on commercial farms (11). Tail lesion estimates have been recorded at abattoirs between 13–72% (12–14), demonstrating the varying prevalence of TB on commercial farms.

Tail-biting has the potential to rapidly increase in frequency and result in cannibalism at the pen or barn level, therefore, early identification and prevention is critical. A common prevention strategy to decrease TB instances is the practice of tail docking pigs soon after birth (11, 15). This practice has been banned in the European Union, yet several member states continue to practice tail-docking due to the continued presence and economic impact of TB (3). The procedure of tail docking is painful (16, 17) and does not necessarily eliminate TB at a later stage of production (18, 19), although tail docking has been demonstrated to be effective at decreasing TB behavior (15, 20, 21). Despite ongoing management efforts, EB and TB episodes are still common and further research exploring alternative avenues of reducing abnormal behavior are needed, with nutritional interventions being of increasing interest (7). Nutritional imbalance in the pig's diet, coupled with modern genetics which result in pigs which grow quickly and have large appetites, may exacerbate the frustration of commercially raised pigs and contribute to EB and TB.

Tryptophan (Trp) is an indispensable amino acid (AA) provided by protein-rich ingredients in the pig's diet or supplemental Trp. Dietary Trp increases plasma Trp (22) and impacts the synthesis of the neurotransmitter serotonin (5-hydroxytryptamine, 5-HT) (7, 23) when fed at levels exceeding dietary requirements on a standardized ileal digestibility (SID) basis. Eating patterns, temperament, sleep cycles, and behavior are all impacted by 5-HT (24). Large neutral amino acids (LNAA) use the same transporter as Trp to enter the brain by crossing the blood brain barrier (23, 25); thus, increasing the quantity of Trp in the diet relative to LNAA increases the transport of Trp across the blood brain barrier (25). The increase in Trp entering the brain leads to an increase in the synthesis of brain 5-HT (25) and this increase may lead to reductions in aggressive tendencies and abnormal behavior in pigs.

Two separate randomized block design (RBD) studies were conducted in the winters of 2018 and 2019 in Ontario, Canada. The objectives of the first and second studies were to investigate the effects of varying levels of dietary Trp, below, at, or above National Research Council (NRC 2012) (26) requirements, on behavior and plasma Trp:LNAA ratio in conventional grower pigs at a high-health herd research facility. We had the following three hypotheses: (1) that plasma Trp concentrations would increase in pigs fed the supplemental diets and decrease in pigs fed the deficient diet relative to the pigs fed the control diets; (2) that the Trp:LNAA ratio would be higher in pigs receiving dietary Trp above recommended NRC 2012 requirements compared to the pigs receiving the control, adequate or deficient diet; and (3) that pigs receiving the supplemented Trp diets would display less aggression and abnormal behavior relative to the pigs receiving the control or deficient diets.

## Materials and Methods

The University of Guelph Animal Care Committee approved all procedures for these studies (Animal Use Protocol #3806).

### Animals and Housing

In study #1, a total of 90, terminal cross Yorkshire x Landrace x Duroc, 8-week-old pigs, from the University of Guelph Arkell Swine Research facility in Guelph, ON, Canada were used in this study. A total of 45 males and 45 females were enrolled over two separate, consecutive trials in study #1 (each separate trial enrolled 45 pigs). For each separate trial in study #1, 80 pigs were initially selected and individually ear tagged. The unique identification numbers of each pig were then entered into a random number generator (random.org) to produce a randomized list of pigs for each trial. The first 45 pig numbers itemized on the randomized list were initially enrolled into the study, balancing for sex to achieve a 50% male and 50% female cohort over the entire study. The pigs were then blocked and allocated to one of nine pens (five pigs per pen over two trials) based on litter origin and sex. All pigs enrolled in the studies had undergone conventional processing within 4 days after birth: tail-docking, teeth-clipping, iron injection, and, if male, castration. Any pigs with an intact tail or intact testes in study #1 were excluded from the study and replaced with the next acceptable pig on the list. < 24-h after introduction to study #1, two pigs were replaced; one was determined to be an intact male, and the other died unexpectedly. The two subsequent pigs on the randomized list were used as replacements. On day 13 of the first trial in study #1, an additional pig was found dead; a post-mortem examination was not conducted. This pig was not sampled for plasma and serum analysis; weight at the time of death was recorded, and all future observations for this pig were recorded as missing in the analysis.

In study #2, a total of 108 pigs of the same breed and age as the pigs in study #1 were used, and the same enrollment protocols were followed. A total of 54 males and 54 females were enrolled over two consecutive trials in study #2. In contrast to study #1, all pigs enrolled in study #2 had intact tails and one pen did have two littermates due to pig deaths during the first trial. On day 10 of the first trial in study #2, a pig was found dead; no post-mortem examination was conducted. This pig had been sampled for plasma and serum analysis, and all future observations for this pig were recorded as missing in the analysis.

The room contained nine separate raised deck pens, each having a dimension of 1.09 m x 2.95 m (3.22 m^2^), with fully slatted floors and metal spindle pen dividers. Each pen housed five pigs in study #1 and 6 pigs in study #2, with a stocking density of 0.64 m^2^/pig and 0.54 m^2^/pig, respectively. The room had been previously cleaned and dried, with an initial temperature of 20°C which was lowered to 19°C on day 11 in study #1, while the temperature remained at 17°C for the duration of study #2. All pens were equipped with a point enrichment device (white plastic object attached to a metal chain) hanging from the ceiling, which was accessible to the pigs in the pen. The scheduled lights in the trial room were activated at 05:30 and turned off at 18:30, ensuring that the pigs received 13 h of light and 11 h of darkness each day for the duration of the trial. Feed and water were provided *ad libitum* to each pen. Each pen had two nipple drinkers available and one dry-feeder trough allowing three pigs to eat simultaneously.

### Diets and Experimental Design

A control (Ctl) diet (Table 1) was commercially prepared and formulated to provide 100% SID requirement of dietary Trp. Two other supplemented diets were formulated; for the supplemented diet (S), Trp was added to provide 175% SID dietary Trp, and for the supplement-plus diet (S+) Trp was added to provide 250% SID dietary Trp. All diets met or exceeded all NRC 2012 (26) nutrient recommendations for grower pigs. All feed was prepared by the University of Guelph, Arkell Feed Mill.

**Table 1:** Diet composition^1^ (% as is basis) of the control (Ctl) and adequate control (AC) pig diets in study #1 and study #2, respectively.

**Nutrient contents**	**Analyzed content**	
	**Ctl diet**	**AC diet**
Metabolizable energy, kcal/kg	3300.00	3300.00
Dry matter, %	88.76	87.29
Crude protein, %	17.23	15.40
Crude fat, %	3.54	3.58
Crude fiber, %	2.33	2.13
Histidine, %	.	0.38
Isoleucine, %	0.69	0.59
Leucine, %	1.51	1.36
Lysine, %	1.18	1.16
Methionine, %	0.34	0.38
Phenylalanine, %	.	0.71
Threonine, %	0.70	0.69
Tryptophan, %	0.19	0.20
Valine, %	0.78	0.74

1 *Ingredient composition: ground corn, soybean meal, tallow, and Bioforce Hog 32*.

On day 1 of study #1 all pens were acclimated to the Ctl diet for the first 7 days. Beginning on day 8 and for the remainder of each trial (29 days) in study #1, three pens received the Ctl diet, three pens received the S diet, and three pens received the S+ diet.

In study #2, an adequate control (AC) diet (Table 1) was commercially prepared and formulated to provide 105% SID requirement of dietary Trp. The two remaining diets were formulated by adding or decreasing the amount of dietary Trp. The deficient diet (D) was formulated to provide 80% SID dietary Trp, while the extra-Trp diet (ET) provided 130% SID dietary Trp. The AC diet and the ET diet met or exceeded all NRC 2012 (26) recommendations for grower pigs. The D diet had < the recommended levels of dietary Trp; however, all other NRC requirements were met or exceeded. The three different diets in study #2 were fed to the appropriate pens beginning on day 1 and continued to be fed for the duration of each trial (15 days) in study #2. Three pens received the AC diet, three pens received the ET diet, and three pens received the D diet.

Feeding troughs were placed into each pen on day 1 and remained in the pen(s) for the duration of the respective study. Feed was provided *ad libitum*, and troughs were filled on as needed basis. Feed spillage was not accounted for in the estimates of intake and feed intake was determined on a per pen basis, which reflected the amount of feed added to each trough.

### Data Collection

**Figure 1:**
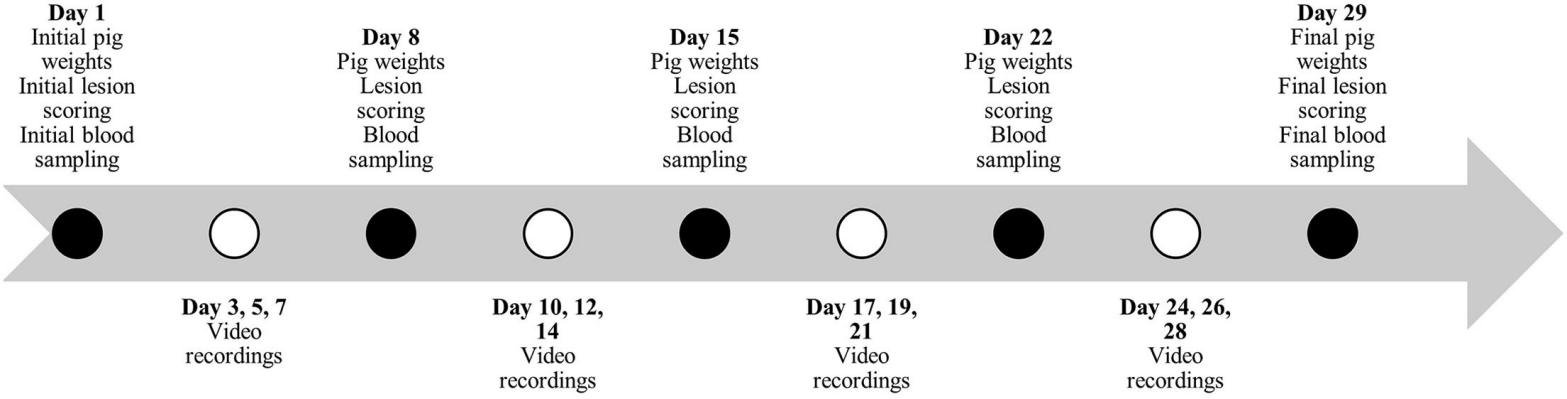
Timeline during study #1 for pig weights, lesion scoring, blood sampling and behavior video recordings.

**Figure 2:**
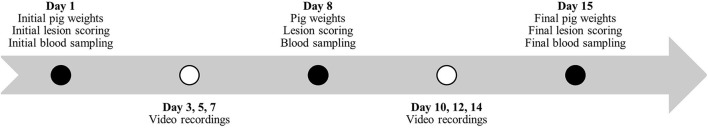
Timeline during study #2 for pig weights, lesion scoring, blood sampling and behavior video recordings.

Individual pig weights and lesion scoring (Table 2) were recorded once every 7 days for the duration of the studies (Figures 1, 2). Lesion scores, including tail, ear, and body scratch scores (Table 2), were recorded by a single observer and all lesion scoring was based on Kritas and Morrison (27) and conducted by the same observer for the duration of the trial. Lesion score recordings were completed before pigs were weighed or blood sampling occurred.

**Table 2:** Tail, ear, and body lesion severity score reference sheet used for study #1 and study #2. Adapted from Kritas and Morrison (27).

**Description**		
**Score**	**Body lesion severity**	**Tail and ear lesion severity**
0	No skin injuries	No evidence of biting
1	Minor skin injuries (occurring sporadically)	Healed or mild lesions
2	Middle skin injuries (occurring over the whole body, no widespread accumulation of injuries)	Evidence of chewing or puncture marks, no swelling
3	Strong skin injuries (several lesions occurring over the whole body, with accumulation of injuries at different areas on the body)	Evidence of chewing or puncture marks, with swelling and signs of infection
4	Very strong skin injuries (lesions densely covering the whole body)	Partial or total loss of the appendage

Plasma and serum samples were collected at 07:30 on allotted days (Figures 1, 2). Blood samples were taken from the orbital sinus of the same three pigs/pen (*N* = 54/study) for the duration of the studies; pigs that were blood sampled were chosen randomly on day 1 of each study; however, sampling included equal numbers of males and females (*N* = 27/study). The blood sampling procedure was performed with the pig manually restrained in dorsal recumbency in a v-trough following the technique described by Huhn et al. (28) using a 16 G, 1.5-inch (1.6 mm x 40 mm) BD Precision Glide^TM^ Needle with 6.0 mL BD Vacutainer^®^ K2 EDTA blood collection tubes for plasma analysis and 10.0 mL BD Vacutainer^®^ serum collection tubes for serum analysis. Blood was collected and stored in a chilled cooler for a minimum of 2 h prior to being centrifuged. Blood was centrifuged at 2,500 rpm for 15 min and within 4 h of collection. Serum aliquots were collected into individual 2 mL micro-centrifuge tubes and labeled according to pig, collection number, trial number, and study number, then placed into −20°C freezer for subsequent AA analysis. Serum samples from sample day 1 and day 29 and day 1 and day 15 were analyzed for Trp, LNAA and the Trp:LNAA ratio for study #1 and #2, respectively. Analysis for AA was conducted using an ultra-performance liquid chromatography system (UPLC; Waters Corporation, Milford, MA). Briefly, 100 μL of 10% sulfosalicylic acid (Sigma Aldrich, Oakville, ON) was added to 100 μL of serum, and then centrifuged using a Fisherbrand accuSpin Micro 17 (Thermo Fisher Scientific, Waltham, MA) at 12,000 rpm for 5 min. Standards for AA and serum were derivatized using an AccQ-Tag Ultra derivatization kit (Waters Corporation, Milford, MA, USA). Derivatized AA were separated and preserved at 55°C using UPLC (Waters Corporation, Milford, MA) with ultraviolet exposure at a wavelength of 260 nm. Amino acid peaks were compared with the AA standards and analyzed using Waters Empower 2 Software (Waters Corporation, Milford, MA, USA). For a complete description, see Templeman et al. (29).

Continuous video recordings for behavior analysis began on day 3 for each study. Five cameras (1 Sony DCRSR-47 and 4 Canon Vixia HF800) were set up on 182.9 cm tripods (Professional Tripod) and placed in the study room to record behavior for 12 h/day, 3 days/week (Figures 1, 2) for the duration of the studies. Identifying numbers on the pigs were refreshed as needed, prior to video recording, and cameras began recording between 05:40 – 06:00. The video observer was not blinded as to which pen was being recorded and analyzed, or to which diet the pen was receiving. Cameras completed recording at 18:00. Analysis of behavior using an ethogram (Table 3) was performed for three specific time periods, chosen for practicality, using the continuous recordings as 10-min scan samples for the following periods: 06:00 – 08:00, 11:00 – 13:00 and 16:00 – 18:00.

**Table 3:** Ethogram used for behavior analysis for study #1 and study #2.

**Behavior**	**Description**
Active	Standing, walking, or sitting without interaction with another pig
Belly nosing	A repeated rhythmic up-and-down massage of the snout on a pen-mate's mid section (30)
Pen-mate nosing	Nosing, sucking, chewing, or biting another pig in an arrhythmic manner (distinct from belly-nosing) (30)
Nosing pen	Manipulating with snout any part of the pen (e.g., pen dividers, floor)
Tail-biting (TB)	Taking the tail of another pig into the mouth
Ear-biting (EB)	Taking the ear of another pig into the mouth
Non-aggressive interaction	Directing investigatory behavior toward another pig, but excluding the performance of other behavior (e.g., sniff/flank-directed)
Aggressive interaction	Engaging in agonistic interaction – pushing, biting, and head-knocking with another pig
Flank-directed oral/nasal	Oral/nasal attention (including bites) to the flank of another pig
At drinker	Drinking water from the nipple drinker
At feeder	Head positioned inside the feeder
Lying	Weight and body not supported by legs, but excluding performance of any of the above behavior
Out of view	Not in view of the camera

### Statistical Analysis

All statistical analysis was performed using SAS (v. 9.4; SAS Institute Inc., Cary, NC).

A mixed effects model was used with diet, sex, and sampling day treated as fixed effects and pen as a random effect. When fixed effects or interactions were not significant, they were removed from the model. A mixed procedure with a maximum likelihood estimation method was used for feed intake, pig body weight, Trp, LNAA and Trp:LNAA analysis.

A GLIMMIX procedure with a binary distribution and a logit link function was used for body and ear lesion scores, with diet (Ctl, S, and S+), sex, and sampling day treated as fixed effects for study #1 analysis. Low occurrences of severity scores above score 1 (Table 2), resulted in a binary outcome of 0 or 1 (for any score equal to or > 1). Body and tail lesion scores were analyzed using a GLIMMIX procedure with a Poisson distribution and a log link function, with diet, sex, and sampling day as fixed effects for study #2. Severity scores (Table 2) were converted to equivalent counts for use in the Poisson model, and a Chi-square exact test was conducted to examine the relationship between ear lesion scores and sex in study #2.

The behavior analysis was performed using a GLIMMIX procedure with a binomial distribution and a logit link function in SAS. A mixed effects model was used with diet, sex, and sampling day treated as fixed effects and pen as a random effect. Non-significant interactions were removed from the model. The mutually exclusive categories of the ethogram (Table 3) for the behavior analysis did not have enough power to use all behaviors. In study #1, the ethogram was condensed to two behaviors: lying or active, with lying as the referent. Lying behavior remained consistent with the definition provided in the ethogram (Table 3), and all other behaviors recorded were considered as active. Lying behavior, active behavior, and aggressive behavior were included in the analysis as outcome variables in study #2.

## Results

### Feed Intake and Weight

In study #1, pigs had a significantly lower feed intake on day 8 as compared to day 15, 22, and 29 (*P* < 0.0001); however, no differences with respect to diet affecting feed intake was detected (*P* > 0.05). The weight of the pigs increased 24.78 ± 2.95 kg and 11.88 ± 2.82 kg over study #1 and #2, respectively (*P* < 0.0001). No differences with respect to diet affecting weight were detected (*P* > 0.05) in either study #1 or study #2. In study #2, an interaction between diet^*^sex (*P* = 0.017) was detected. Female pigs fed the AC diet (30.61 ± 6.35 kg) were significantly heavier compared to males fed the AC diet (27.61 ± 5.87 kg) (*P* = 0.008), males fed the D diet (27.82 ± 5.87 kg) (*P* = 0.014), females fed the D diet (27.26 ± 5.73 kg) (P = 0.003), and females fed the ET diets (27.27 ± 6.07 kg) (*P* = 0.003). During study #2, greater amounts of feed were added to each pen between day 1 and day 8 of the study, compared to day 8 to day 15 (*P* < 0.0001); however, no differences with respect to diet were detected (*P* > 0.05).

### Lesion Scores

In study #1, there was a significant difference in recorded body lesions based on sampling day (*P* < 0.0001); however, no differences due to diet or sex were detected. The odds of having a body lesion recorded on day 1 was 5.28 (95% CI: 2.92–9.56) times > on day 8 and 1.86 (95% CI: 1.02– 3.40) times > on day 29. There were significant differences in ear lesion scores based on day of the study (*P* < 0.0001); however, no differences due to diet or sex were detected. The odds of having an ear lesion recorded on day 1 was 8.85 (95% CI: 4.27–18.36) times > on day 8; 7.97 (95% CI: 3.89–16.33) times > on day 15; 16.59 (95% CI: 7.03–39.17) times > on day 22; and 35.10 (95% CI: 11.63–105.95) times > on day 29. The odds of having an ear lesion recorded on day 8 was 3.97 (95% CI: 1.24–12.69) times > on day 29. The odds of having an ear lesion recorded on day 15 was 4.40 (95% CI: 1.39–13.99) times > on day 29. No tail lesions were recorded during study #1.

In study #2, no relationship between the incidence of ear lesion scores and diet or sex was detected (*P* > 0.05). No differences between diets, sex or sampling day were detected for body lesion scores. A sex^*^sampling day interaction was significantly impacting (*P* = 0.015) the tail lesion score recorded. On day 1, females had a 6.8 (95% CI: 2.18–20.88) times higher risk of having a tail lesion recorded than males (*P* = 0.001); females had a 3.7 (95% CI: 1.52–8.88) times higher risk of tail lesions on day 1 compared to day 15 (*P* = 0.004) and a 3.4 (95% CI: 1.55– 7.48) times higher risk of tail lesions on day 8 compared to day 15 (*P* = 0.003). A protective effect in males for tail lesions was detected on day 1 compared to day 8 (*P* = 0.01) (Table 4).

**Table 4:** Significant risk rate of pigs having a tail lesion score recorded (based on the severity score sheet, adapted from Kritas and Morrison (27)) by sample day of study #2.

**Effect**	**Risk rate**	**Confidence interval**	***P-*value**
Day 1 vs. Day 15 (females)	3.67	1.52 – 8.88	**0.004**
Day 1 (females vs. males)	6.75	2.18 – 20.88	**0.001**
Day 8 vs. Day 15 (females)	3.40	1.55 – 7.48	**0.003**
Day 1 vs. Day 8 (males)	0.25	0.09 – 0.71	**0.010**

### Behavior

In the fixed effects model in study #1, no statistical differences were found for pigs performing lying behavior or active behavior based on diet, sex, and sampling day (*P* > 0.05). In study #2, an interaction between diet^*^sampling day of the study (*P* = 0.02) was significant for pigs performing active behavior. The odds of pigs performing active behavior, while being fed the AC diet, was 1.59 (95% CI: 1.10–2.31) times greater on day 5 and 1.55 (95% CI: 1.02–2.34) times greater on day 12 compared to pigs receiving the ET diet on the same days. No differences were detected between the diets fed or sex with respect to lying behavior; however, the odds of pigs performing lying behavior varied by sampling day. The odds of pigs performing lying behavior on days 3, 5, 7, 10, and 12 were > on day 14 (*P* < 0.0001) (Table 5). No differences were detected between the diets fed or sex with respect to aggressive behavior; yet significant differences did exist between the odds of performing aggressive behavior between specific sampling days. The odds of pigs performing aggressive behavior was greater on day 5 (5.69, 95% CI: 2.38–13.58) and day 7 (4.11, 95% CI: 1.68–10.06) compared to day 10 (Table 5).

**Table 5:** Significant odds ratios (OR) of pigs performing lying and aggressive behavior (based on the ethogram) by sample day of study #2.

**Effect**	**Odds Ratio (OR)**	**Confidence interval**	***P*-value**
**Lying Behavior**			
Day 3 vs. Day 10	0.80	0.73 – 0.89	<0.0001
Day 3 vs. Day 14	1.30	1.19 – 1.43	<0.0001
Day 5 vs. Day 10	0.83	0.76 – 0.92	0.0002
Day 5 vs. Day 12	1.11	1.01 – 1.22	0.031
Day 5 vs. Day 14	1.35	1.23 – 1.48	<0.0001
Day 7 vs. Day 10	0.80	0.73 – 0.88	<0.0001
Day 7 vs. Day 14	1.29	1.18 – 1.42	<0.0001
Day 10 vs. Day 12	1.33	1.21 – 1.47	<0.0001
Day 10 vs. Day 14	1.62	1.47 – 1.78	<0.0001
Day 12 vs. Day 14	1.21	1.11 – 1.33	<0.0001
**Aggressive Behavior**			
Day 3 vs. Day 5	0.33	0.17 – 0.65	0.001
Day 3 vs. Day 7	0.45	0.22 – 0.45	0.029
Day 3 vs. Day 14	0.37	0.19 – 0.37	0.005
Day 5 vs. Day 10	5.69	2.38 – 13.58	<0.0001
Day 7 vs. Day 10	4.11	1.68 – 10.06	0.002
Day 10 vs. Day 12	0.29	0.12 – 0.71	0.007
Day 12 vs. Day 14	0.20	0.08 – 0.48	0.0003

### Trp, LNAA, and Trp:LNAA

Plasma Trp concentrations were significantly different between diet and sampling day (interaction *P* < 0.0001) in study #1. The pigs fed the S+ diet had greater concentrations of plasma Trp (182 ± 42 μmol) compared to the pigs fed the S diet (132 ± 40 μmol) and pigs fed the Ctl diet (89 ± 20 μmol) on day 29 compared to day 1. Diet, sex and sampling day did not impact the LNAA concentration. The ratio of Trp:LNAA was significant between diet and sampling day (interaction *P* < 0.0001). Significantly higher ratios of Trp:LNAA were detected in the pigs fed the S+ diet (0.18 ± 0.03) and the S diet (0.13 ± 0.03) compared to pigs fed the Ctl diet (0.09 ± 0.01) between day 1 and day 29. In contrast to study #1, no statistical differences were detected between diet or sex for plasma Trp, plasma LNAA, or the Trp:LNAA ratio (*P* > 0.05) in study #2.

## Discussion

The data obtained from the two studies suggest that varying levels (deficient, adequate, and supplemental) of dietary Trp does not alter behavior in a high-health herd with minimal challenges. Regardless of the allocated dietary treatment, the pigs demonstrated low levels of aggression, abnormal behavior, and activity, in relation to lying behavior. The sample size for each study was based on previous work by Poletto et al. (31), which demonstrated that high-Trp diets reduced agonistic and aggressive interactions in three-month-old gilts. Experimental diets used included supplemental and deficient dietary Trp; however, differences between the control diets and the experimental diets were not detected when investigating feed intake, body weight, bite lesions, or behavior.

Ear, tail, and body lesions were observed throughout the studies, although no severe lesion scores (Score 4) were recorded; the lesions were equally distributed between the diets and pens. Lesion score is associated with bouts of aggression and agonistic episodes and may be a proxy for characterizing dominance in hierarchy formation of pigs (32). The low occurrences of lesions and aggression within the pens, suggested that maintaining or acquiring dominance was not a significant factor during the studies. No tail damage was recorded throughout the first study, which may have been the result of tail-docking (15, 20, 21). In the second study, tails were intact, and therefore longer, which may account for the TB instances recorded. Females had a higher risk of having tail damage compared to males during the first two sampling days in the second study, but not on the final sampling day. It has been suggested that females are more likely to perform TB behavior and also receive TB wounds (33) in both all-female and mixed-sex pens. Pigs that engage in TB are also known to engage more frequently in other oral abnormal behavior, such as EB and FB (8).

The ability of Trp to cross the blood brain barrier relies on the ratio of Trp:LNAA in the diet (25), as higher circulating levels of plasma Trp relative to the concentration of LNAA, are able to cross the blood brain barrier more effectively (25). A period of acclimation to the diets may be necessary (34) to observe an increase in plasma Trp and observe the behavioral modifications related to dietary Trp intake and plasma 5-HT concentrations (7, 34). Koopmans et al. (34) suggested an acclimation period of at least 5– 7 days in pigs; however, the period of time necessary to detect changes in Trp, 5-HT and behavior, appears to differ between monogastric species (29, 34). In the first study, all pigs were habituated to the Ctl diet for 7 days prior to the experimental diets being fed; pigs fed the S+ diet had significantly greater concentrations of plasma Trp in contrast to pigs fed the Ctl diet or the S diet for 21 days. During the second study, pigs were fed the assigned diets (D, AC, and ET) for the duration of the trial (15 days), and differences in Trp, LNAA, and Trp:LNAA were not detected between the three experimental diets. The results suggested that pigs housed in similar circumstances to the current studies require a period of analysis longer than the previously stated 5 – 7 days to observe changes in plasma Trp, the ratio of Trp:LNAA and the possible corresponding behavioral modifications which may occur due to varying levels of dietary Trp.

When dietary Trp is supplied at levels under known population requirements, pigs will experience reduced feed intake and growth (23, 24, 35). Pigs are able to self-identify a diet under supplied in Trp and have an aversion to ingesting the deficient feed (36). The results of the second study suggested that the D diet may not have been Trp-deficient enough or was not fed for a long enough duration to observe feed intake and growth differences in the study population. Sex differences exist for Trp and 5-HT requirements as gilts have been found to have a greater susceptibility to an imbalance in Trp:LNAA compared to barrows (23, 35). Although the current studies did not demonstrate this, it is possible that a longer period of time may have been necessary to truly demonstrate the sex differences with respect to the Trp:LNAA ratio. Dissimilar phenotypes of pigs in a TB event (biters, victims or neutral pigs) have been found to have differing levels of 5-HT (37). Pigs which perform and are victims of TB have lower platelet 5-HT than neutral pigs (37), suggesting that both victims and biters require increased dietary Trp to increase 5-HT in the body. The current studies displayed few instances of aggression or abnormal behavior compared to lying behavior, and all pigs had appropriate feed intake, and gained weight as expected for the facility and the stage of production, regardless of the diet.

A limitation during the studies was the low levels of abnormal behavior observed during the period of data collection. Studies have demonstrated that differences in Trp metabolism were detected when pigs were challenged by disease (5, 38–41) therefore, future studies should investigate the optimal concentration of indispensable AA at each stage of production to modify behavior when an immune challenge or abnormal behavior is present. The diets for the second study may not have been fed for a long enough duration to truly impact behavioral or physiological changes in the pig (29, 34). Pigs were not balanced into feeding treatments based on weight, as well, no blinding as to which diet the pigs were receiving was undertaken, which could have impacted the objectivity of both the diet and the behavior analysis. However, no diet-related effects on growth or behavior were observed, suggesting that this was not an issue. Future research should include Trp supplemented diets as an intervention in commercial barns with on-going cannibalistic issues to fully assess the usefulness of the AA in a stressful, conventional environment.

## Conclusion

Low levels of aggression and biting behavior in the barn led to challenges when attempting to detect differences in the behavior of pigs based on diet. No differences were detected between the diets when assessing growth, feed intake, ear-biting, tail-biting, body scratch scores, or behavior for the duration of the studies, yet plasma Trp and the Trp:LNAA ratio were different based on diet in study #1. This suggests that an increase in dietary Trp levels can impact both circulating plasma Trp, and 5-HT concentrations in the pig, but that these differences alone do not impact ear-biting, tail-biting and agonistic behavior.

## Data Availability Statement

The raw data supporting the conclusions of this article will be made available by the authors, without undue reservation.

## Ethics Statement

The animal study was reviewed and approved by the University of Guelph Animal Care Committee (Animal Use Protocol #3806).

## Author Contributions

Data analysis by MH with recommendations by TO'S, LN, AS and RF. Manuscript written by MH with contributions from TO'S, LN, AS and RF. All authors contributed to the article and approved the submitted version.

## Funding

Funding was provided by the Ontario Agri-Food Research Innovation Alliance, the Ontario Veterinary College Departmental Graduate Growth Support Stipend, and Grand Valley Fortifiers (Cambridge, ON, Canada).

## Conflict of Interest

The authors declare that the research was conducted in the absence of any commercial or financial relationships that could be construed as a potential conflict of interest.

## Publisher's Note

All claims expressed in this article are solely those of the authors and do not necessarily represent those of their affiliated organizations, or those of the publisher, the editors and the reviewers. Any product that may be evaluated in this article, or claim that may be made by its manufacturer, is not guaranteed or endorsed by the publisher.

## Abbreviations

°C: degrees Celsius
μmol: millimole
5-HT: serotonin
AA: amino acid
AC: adequate control diet
ANOVA: analysis of variance
CI: confidence interval
cm: centimeters
Ctl: control diet
D: deficient diet
EB: ear-biting
ET: extra-tryptophan diet
FB: flank-biting
kcal: kilocalorie
kg: kilogram
LNAA: large neutral amino acid
m: meter
mL: milliliter
mm: millimeter
NRC: National Research Council
OR: odds ratio
RBD: randomized block design
RPM: revolutions per minute
S: supplemental diet
S+: supplement plus diet
SD: standard deviation
SID: standard ileal digestible
TB: tail-biting
Trp: tryptophan.
